# Catalase Specifically Binds Antipsychotic Clozapine: Experimental and In Silico Insights into Interactions, Complex Stability, and Dose-Dependent Enzyme Activity Modulation

**DOI:** 10.3390/molecules31081294

**Published:** 2026-04-16

**Authors:** Tamara Vasović, Milica Radibratović, Dušan Spasić, Simeon Minić, Čedo Miljević, Nikola Gligorijević, Milan Nikolić

**Affiliations:** 1 Department of Biochemistry, Faculty of Chemistry, University of Belgrade, 11000 Belgrade, Serbia; tvasovic@chem.bg.ac.rs (T.V.); sminic@chem.bg.ac.rs (S.M.); 2 Department of Chemistry, Institute for Chemistry, Technology and Metallurgy, National Institute of the Republic of Serbia, University of Belgrade, 11000 Belgrade, Serbia; milica.radibratovic@ihtm.bg.ac.rs; 3 Department of Pharmacology, Clinical Pharmacology and Toxicology, Faculty of Medicine, University of Belgrade, 11000 Belgrade, Serbia; dusan.spasic.dr@med.bg.ac.rs; 4 Institute of Mental Health, Faculty of Medicine, University of Belgrade, 11000 Belgrade, Serbia; cedo.miljevic35@gmail.com

**Keywords:** clozapine, catalase, binding interactions, enzyme structure, enzyme activity

## Abstract

Oxidative stress is intrinsically linked to mental disorders, involving an imbalance between reactive species and antioxidant defenses, where catalase is an essential, ubiquitous antioxidant enzyme. The pleiotropic effects of antipsychotic drugs, used for schizophrenia and mood disorders, are not fully elucidated at the molecular level. This study characterized the binding of a highly effective but potentially dangerous antipsychotic, clozapine (CLZ), to commercial bovine liver catalase (BLC). Using various spectroscopic methods under simulated physiological conditions, we found a moderate binding affinity of CLZ for BLC (*K*_a_ = 1.4 × 10^−5^ M^−1^), subtly influencing the protein’s secondary and tertiary structures and slightly increasing its thermal stability. CLZ efficiently protected BLC against free-radical-induced oxidation and preserved its catalytic activity for decomposing toxic hydrogen peroxide. The effect of CLZ on BLC antioxidant activity was dual: no significant effect at lower, physiologically relevant concentrations, but significant inhibition at saturating, toxic drug concentrations. Molecular docking and molecular dynamics results indicated the presence of two specific binding sites within BLC monomers, one located near its active site. In conclusion, our in vitro results indicate that CLZ’s specific binding to BLC can be both beneficial and potentially harmful, and that this effect is dose-dependent.

## 1. Introduction

Clozapine (CLZ), or 8-chloro-11-(4-methyl-1-piperazinyl)-5*H*-dibenzodiazepine, is an atypical antipsychotic drug and the gold standard in the treatment of refractory schizophrenia [1]. Unlike conventional antipsychotics, CLZ exhibits only mild dopamine D2 receptor antagonism, with a shorter duration of occupancy, thereby significantly reducing the occurrence of extrapyramidal symptoms such as tremors, rigidity, and tardive dyskinesia [2]. Moreover, CLZ’s interactions with a variety of neurotransmitter systems, including serotonin (5-HT2A, 5-HT2C), adrenergic (*α*1 and *α*2), histamine (H1), and muscarinic acetylcholine (M1) receptors, contribute to its effectiveness in managing a broad spectrum of schizophrenia symptoms [3]. Serious adverse effects, including agranulocytosis, cardiotoxicity, metabolic syndrome, and gastrointestinal hypomotility, limit its clinical use and necessitate continuous hematological and metabolic surveillance [4]. Therefore, unlike other atypical antipsychotics with broader safety profiles, CLZ uniquely requires lifelong monitoring—a requirement established since its initial regulatory approval and consistently reinforced by preclinical and clinical safety data [5]. For adults over 16 years of age, treatment typically begins with low oral doses of 12.5–25 mg/day and is carefully adjusted to maintain a therapeutic CLZ blood level of 350–600 ng/mL, based on the patient’s response [6].

Oxidative stress, arising from an imbalance between reactive oxygen species (ROS) generation and antioxidant defenses, is strongly implicated in neuronal damage and the pathophysiology of schizophrenia [7]. Although CLZ effectively manages treatment-resistant cases, accumulating evidence indicates it can exacerbate oxidative imbalance by increasing ROS levels and impairing antioxidant enzyme activity, particularly at moderate to high doses [8]. For example, CLZ has been shown to elevate superoxide production in neutrophils, potentially triggering apoptosis and contributing to its association with agranulocytosis [9]. Additionally, bioactivated CLZ metabolites have been reported to deplete intracellular glutathione in hematopoietic cell lines, further disrupting redox homeostasis [10]. Conversely, CLZ also exhibits protective effects against oxidative stress under specific conditions. It has been reported to protect neuron-like rat pheochromocytoma (PC-12) cells from hydrogen peroxide-induced oxidative damage by inhibiting extracellular signal-regulated kinase phosphorylation [11]. The observed protective activity of CLZ against ketamine-induced cell death in adult neural stem cells was associated with increased expression of anti-apoptotic markers, indicating its neurogenesis-promoting activity [12]. Dualistic redox homeostasis effects set CLZ apart from other antipsychotics, underpinning its pharmacodynamic complexity, and may partially explain both its therapeutic efficacy and side-effect spectrum.

Catalase (EC 1.11.1.6), an essential antioxidant enzyme, protects cells from oxidative injury by converting toxic hydrogen peroxide (H_2_O_2_), a byproduct of cellular metabolism, into harmless water and oxygen [13]. Human catalase is a homotetramer (approximately 240 kDa), composed of four identical tetrahedrally arranged subunits, each harboring a ferriprotoporphyrin IX (heme) group and an NADPH molecule. The central structural core of each subunit contains an eight-sheeted anti-parallel *β*-barrel domain linked to a six *α*-helical domain (a “catalase fold”) via a lengthy amino acid sequence [14]. Catalase’s remarkable catalytic efficiency is structurally supported: a heme group is essential for H_2_O_2_ decomposition, tightly bound NADPH acts as a protective agent against inactive enzyme formation, and electrostatic surface properties and specific channels facilitate rapid substrate access, ensuring high turnover. These features are critical for preserving cellular redox homeostasis and protecting macromolecules from oxidative damage during physiological and pharmacological stress [15].

This study, for the first time, characterized interactions between the CLZ drug and the catalase enzyme, using commercial bovine liver catalase (BLC) as a usual model for human catalase due to their similar structure (four identical subunits, 527 amino acids per subunit, each containing a heme prosthetic group), high sequence similarity, and comparable function as primary peroxisomal antioxidants, with minor differences in specific catalytic activity [16]. We used a multi-modal approach, integrating spectroscopic methods, circular dichroism, molecular docking, and extended molecular dynamics (MD) simulations, to elucidate the structural and functional consequences of BLC–CLZ complex formation. Our integrated methodology provides insights into how CLZ modulates catalase conformation, thermal stability, and enzymatic activity. These findings expand the current understanding of CLZ’s redox interactions at the protein level [17,18], contributing to its pleiotropic effects.

## 2. Results and Discussion

### 2.1. Characterization of Clozapine Binding to Bovine Liver Catalase

Standard spectroscopic methods [19] were used to study the formation of protein–ligand (BLC–CLZ) complex, under simulated physiological conditions (pH 7.2, 37 °C).

#### 2.1.1. Protein Fluorescence Quenching Study Results

Fluorescence quenching characterizes protein–ligand binding, quantifying the decrease in protein intrinsic fluorescence as a ligand binds, signaling conformational changes or direct environmental shifts. It enables determination of binding parameters and mechanisms, though it requires correction for inner-filter effects (ligand absorption) to avoid misinterpretation of the obtained data [20].

**Figure 1:**
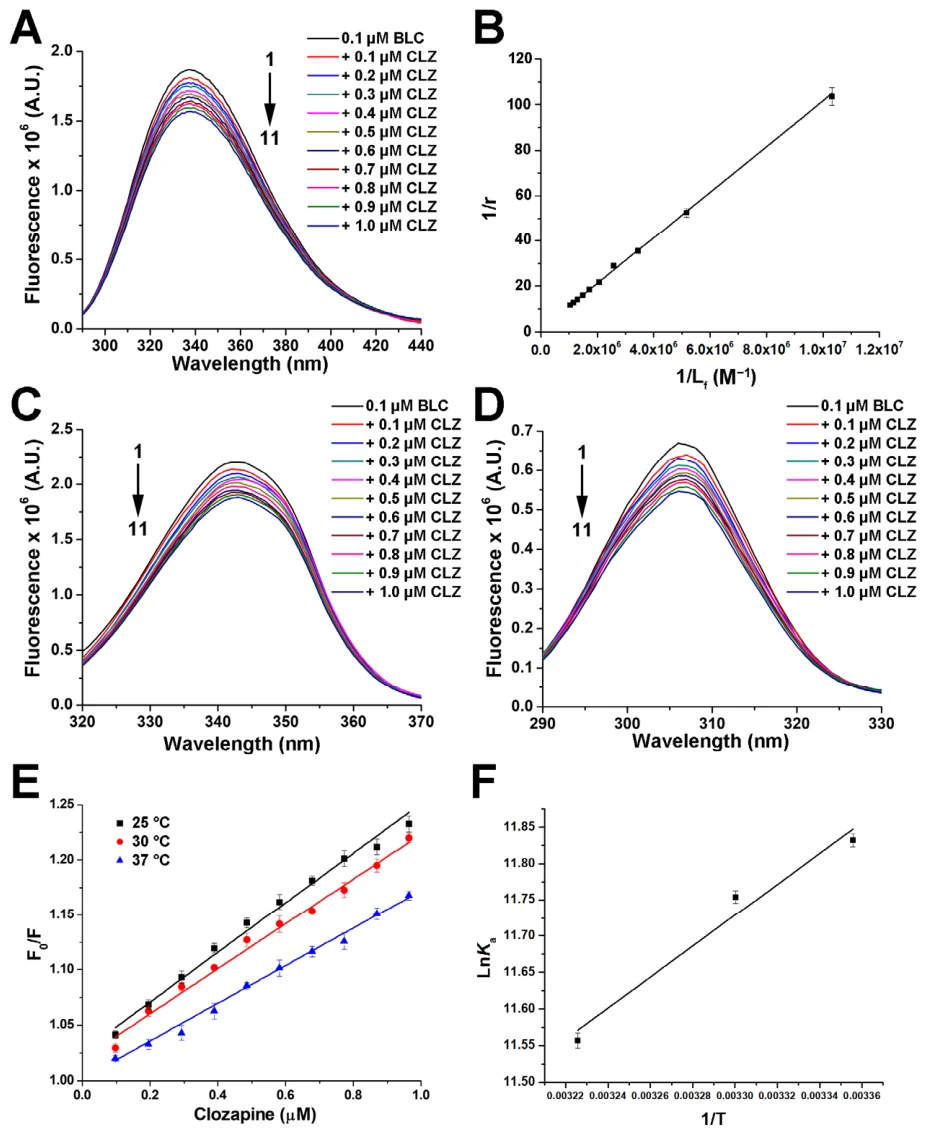
The quenching of bovine liver catalase (BLC) fluorescence by clozapine (CLZ). (**A**) Representative emission spectra (excitation at 280 nm) of BLC (0.1 μM) in the presence of increasing CLZ concentrations (0–1 μM). (**B**) Klotz double reciprocal plot used to calculate the binding (affinity) constant. Representative synchronous fluorescence spectra of BLC (0.1 μM) with (**C**) Δλ = 60 nm (for Trp residues) and with (**D**) Δλ = 15 nm (for Tyr residues) in the presence of increasing CLZ concentrations (0–1 μM). (**E**) Stern–Volmer (SV) plot at 25 °C, 30 °C, and 37 °C, used for the calculation of SV quenching constants (*K*_SV_). (**F**) van’t Hoff’s diagram to calculate thermodynamic parameters of BLC–CLZ complex formation. Error bars (**B**,**E**,**F**) represent the standard deviation (*n* = 3).

BLC emission fluorescence spectra were recorded in the absence and presence of CLZ (up to a protein: ligand molar ratio of 1:10), at three different temperatures (Section 3.2). As shown in Figure 1A, the enzyme exhibited a fluorescent emission peak at 340 nm upon excitation at 280 nm. This fluorescence is primarily due to aromatic amino acid residues, specifically tryptophan (Trp) and, to a lesser extent, tyrosine (Tyr). The BLC amino acid sequence contains 6 Trp and 22 Tyr residues per tetramer [16]. The addition of CLZ dose-dependently reduced the fluorescence intensity, indicating binding interactions between the enzyme and the drug. The fluorescence emission maximum (λ_max_) remained unchanged during quenching. Intensities at the emission maxima, corrected for the inner filter effect, were used to calculate binding affinity. The binding curves (Figure 1B) were obtained from quenching data, and then *K*_a_ was calculated using Equation (2). The binding constants at three different temperatures are shown in Table 1. Obtained values for CLZ binding to BLC range between 1.23 and 1.45 × 10^5^ M^−1^, indicating moderately strong binding and showing a slight decrease with increasing temperature.

Synchronous fluorescence spectroscopy is a sensitive technique for analyzing protein–ligand binding by simultaneously scanning excitation and emission wavelengths with a constant difference (Δλ) [21]. The changes in the synchronous spectra of catalase at Δλ = 15 nm (Tyr) and Δλ = 60 nm (Trp) after incubation with CLZ are shown in Figure 1C,D. In line with the results from classical emission spectra of BLC in the presence of the drug (Figure 1A), the addition of CLZ decreases the fluorescence of both amino acids within the protein. As reported in the literature [22], both Trp and Tyr residues contributed to fluorescence quenching upon ligand binding, but Trp’s overall contribution was more significant. Since the emission maxima positions of both Trp and Tyr remained almost unchanged in the presence of increasing drug concentrations, this indicates that the microenvironment polarity around these amino acid residues within BLC remains the same when CLZ is bound. On the other hand, the binding of antidegradant 2-mercaptobenzimidazole (MBI) to BLC, for example, induces a red shift (an increase in the wavelength of maximum fluorescence emission) of Tyr and a blue shift in Trp residues, implying conformational re-ordering of the protein [23]. The position of a binding site on a protein may significantly alter the amino acid microenvironment. While MBI binds at the interface of chains B and C [23], CLZ preferentially binds within the enzyme monomer’s structure (Section 2.3).

Stern-Volmer plots at three temperatures for the BLC–CLZ interaction displayed linearity across all tested concentrations (Figure 1E), consistent with a single predominant quenching mechanism. Such linearity typically arises from either static quenching (caused by the formation of a non-fluorescent ground-state complex) or dynamic quenching (resulting from collisions between excited-state fluorophores and quenchers) [24]. From the calculated *K*_sv_ values, the bimolecular rate constant (*k*_q_) for CLZ was determined to be in the order of 10^13^ M^−1^ s^−1^ (Table 1), significantly exceeding the 1 × 10^10^ M^−1^ s^−1^ threshold commonly associated with the diffusion-controlled association of two molecules in aqueous solution [25], providing clear evidence that the observed quenching is due to a complex formation between BLC and CLZ.

When compared with literature values for various drugs binding to BLC *via* steady-state fluorimetry at 25 °C, the CLZ value (1.4 × 10^5^ M^−1^) was two orders of magnitude lower than that reported for the binding of the antidiabetic agent pioglitazone (5 × 10^7^ M^−1^) [26]. However, they are comparable to those reported for the antimicrobial agent triclosan (0.5 × 10^5^ M^−1^) [27] and the flavonoid isorhamnetin (0.6 × 10^5^ M^−1^) [28], while exceeding those for the antitubercular drug clofazimine (2.5 × 10^4^ M^−1^) [29], salicylic acid (4.4 × 10^4^ M^−1^) [30], nifedipine, a calcium-channel blocker (1.9–6.7 × 10^4^ M^−1^) [31], and bifendate analogs (0.5–3.0 × 10^4^ M^−1^) [32]. Indeed, BLC can bind a wide range of small bioactive molecules with moderate affinities (often in the 10^4^–10^6^ M^−1^ range), positioning it as another factor in their complex pharmacokinetics (“what the body does to a drug”) and, therefore, pharmacodynamics (“what a drug does to the body”).

#### 2.1.2. Thermodynamics of Bovine Liver Catalase–Clozapine Complex Formation

Calculated thermodynamic parameters for CLZ binding to BLC, obtained from the van’t Hoff plot (Figure 1F), are shown in Table 1. The negative Δ*G* values, ranging from −29.3 kJ mol^−1^ to −29.8 kJ mol^−1^, confirmed that the interaction is spontaneous under the given conditions at all examined temperatures. These Δ*G* values are also consistent with the thermodynamics of moderate-affinity ligand binding, roughly −4 to −9 kcal mol^−1^ or (approximately) −16 to −38 kJ mol^−1^ at room temperature [33]. The Δ*G* value for CLZ binding (cca. −29 kJ mol^−1^) closely matched those previously reported for triclosan [27], suggesting comparable spontaneity in complex formation. In contrast, ligands with higher affinity, such as pioglitazone, typically exhibit more negative Δ*G* values (cca. −40 kJ mol^−1^), reflecting stronger interactions with BLC [26].

The enthalpy change (Δ*H*) of −17.8 ± 0.12 kJ mol^−1^ for CLZ binding to BLC suggests that the interaction is exothermic, with favourable non-covalent interactions, such as hydrogen bonds and van der Waals forces, contributing to drug–enzyme complex formation. The positive entropy change (Δ*S*) of 38.8 ± 0.48 J mol^−1^ K^−1^ indicates that the binding process is also driven by the hydrophobic effect [34]. The thermodynamic profile of CLZ binding to BLC revealed a moderate-affinity interaction, driven primarily by entropy, with some enthalpic contribution. These results are similar to those of other ligands that bind to BLC, particularly those that rely on the hydrophobic effect, such as polychlorinated aromatic ether triclosan [27], 3′-*O*-methylated flavonol isorhamnetin [28], and thiazolidinedione drug pioglitazone [26].

### 2.2. Characterization of the Effects of Clozapine Binding to Bovine Liver Catalase

The influence of the BLC–CLZ complex formation on the enzyme’s structure, stability, and catalytic activity was evaluated using standard optical spectroscopic methods, which provide real-time, non-destructive analysis of the protein’s microenvironment and behavior [19].

#### 2.2.1. Effect of Complex Formation on the Enzyme Structure

**Figure 2:**
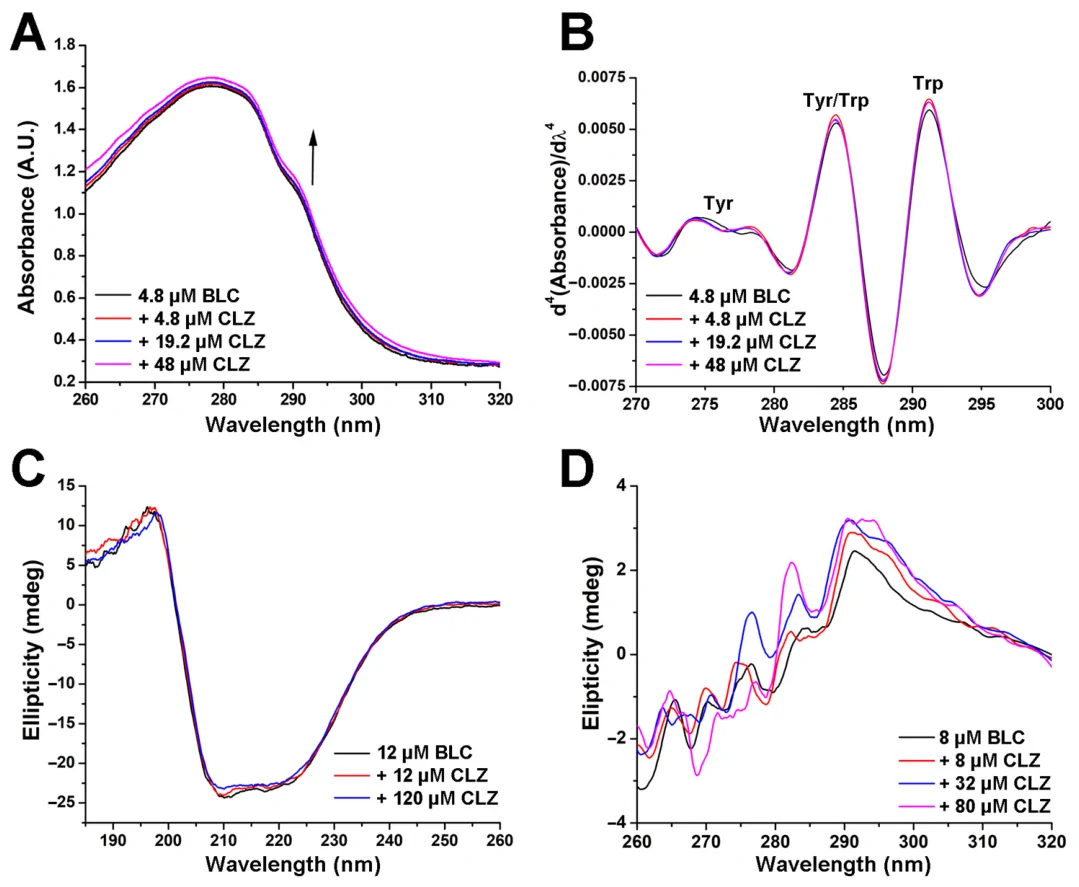
The influence of clozapine (CLZ) binding on bovine liver catalase (BLC) structure. (**A**) UV spectra of BLC (4.8 μM) in the presence of increasing concentrations of CLZ (0–48 μM). (**B**) Fourth derivative of UV absorption spectra of BLC (4.8 μM) in the presence of increasing concentrations of CLZ (0–48 μM). (**C**) Representative far UV-CD spectra of BLC (12 μM) in the presence of increasing concentrations of CLZ (0–120 μM). (**D**) Representative near UV-CD spectra of BLC (8 μM) in the presence of increasing concentrations of CLZ (0–80 μM).

From the BLC’s UV absorption spectra (Figure 2A), fourth-derivative spectra were constructed in the presence of up to a tenfold excess of CLZ (Figure 2B) to better understand the protein structure in the drug complex, resolving overlapping absorption peaks associated with aromatic residues [35]. Three distinct peaks appear, which correspond to Tyr (276 nm), a combination of Tyr and Trp (284 nm), and Trp (292 nm) [36]. Both original (“raw”) and fourth derivative absorption spectra are very similar for pure BLC and in the presence of CLZ, confirming that the microenvironment around both Trp and Tyr remains unaltered after complex formation. This result is in agreement with spectrofluorimetry data. The Soret band of BLC, the most prominent spectral feature in the near-UV/visible region at 406 nm, which corresponds to the heme group within the protein and is responsible for the enzyme’s activity [14], was also unchanged, suggesting the enzyme has not undergone significant structural rearrangement or denaturation upon drug (ligand) binding.

The results presented in Figure 2C and Table 2 illustrate the effects of CLZ on the secondary structure of BLG, as determined using far-UV circular dichroism (CD) spectroscopy. The far-UV CD spectrum of the pure enzyme exhibited a characteristic α-helical shape, with two negative bands at 209 and 220 nm [37]. CD-pro tool, a comprehensive free software package [38] for determining secondary structure fractions from far-UV CD spectra, revealed the expected total content of ordered structural elements (approx. 60%) in the native commercial BLC form. CLZ binding induced subtle alterations in the secondary structure of BLC. When the drug was introduced at a 1:1 molar ratio, the *α*-helical content of catalase decreased slightly while the *β*-sheet content increased. At a higher molar ratio of 1:10 (drug in excess), the same trend continued (Table 2). This alteration suggests that the binding of CLZ induced fine conformational rearrangements in the protein, potentially stabilising *β*-sheet structures at the expense of the *α*-helices. Minor but consistent alterations in BLC’s secondary structure upon CLZ binding suggest an induced-fit mechanism, in which localized conformational adjustments optimize protein–ligand interactions, thereby increasing binding affinity and specificity [39]. This interpretation aligns with prior findings showing that triclosan binding to BLC decreased *α*-helical content and increased *β*-sheet content [27]. In contrast, studies of pioglitazone binding to this essential, highly active antioxidant enzyme indicated a more pronounced stabilisation of *α*-helices [26], highlighting the ligand-specific effects on catalase’s secondary structure.

Near-UV CD spectroscopy showed that the binding of CLZ to BLC resulted in more noticeable alterations in the near-UV CD spectrum (Figure 2D). The addition of this antipsychotic drug increased the near-UV CD signal in the area of Trp residues. This increase is more significant at higher CLZ concentrations. The areas belonging to Phe and Tyr residues remain almost unaltered. The results indicate that the structural rigidity of the enzyme’s asymmetric regions surrounding Trp increased upon ligand binding [40], confirming subtle changes in the BLC structure upon CLZ binding.

#### 2.2.2. Effect of Complex Formation on Bovine Liver Catalase Thermal Stability

**Figure 3:**
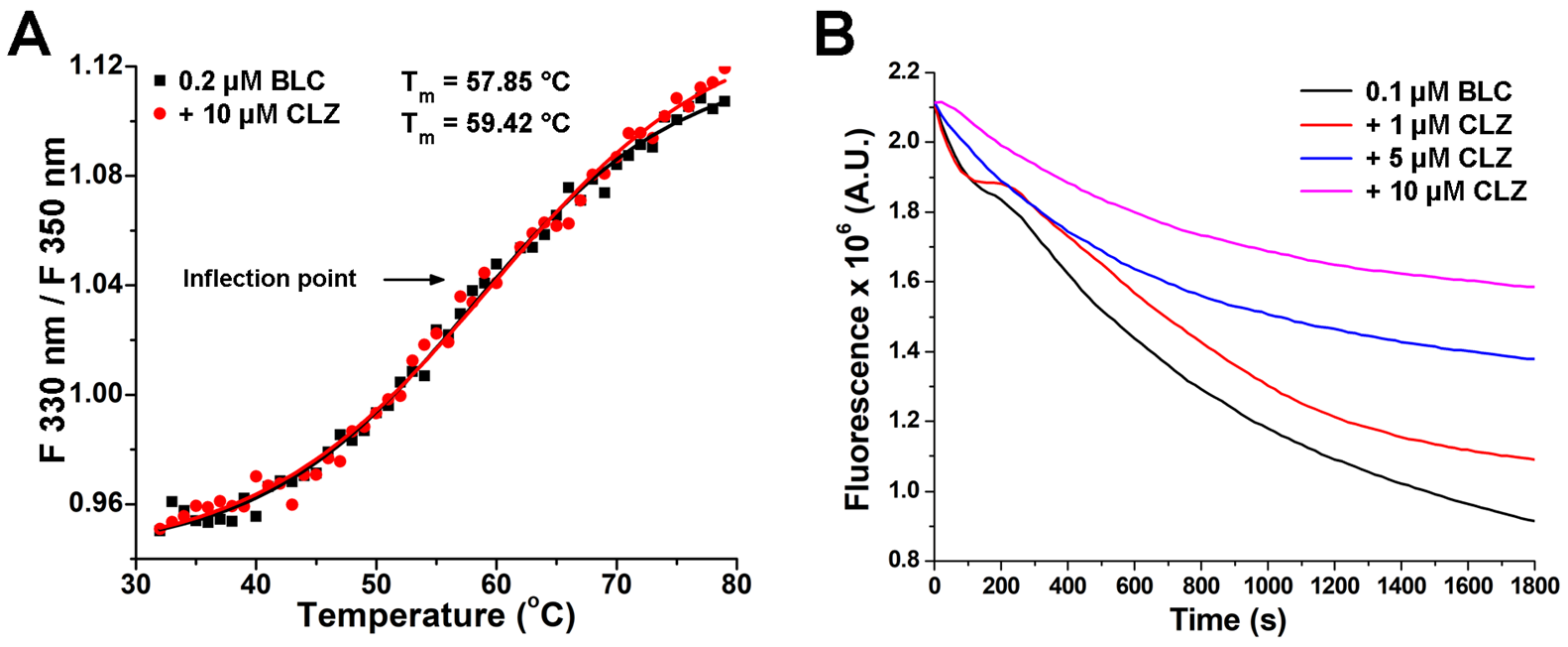
The influence of clozapine (CLZ) on thermal and oxidative stability of bovine liver catalase (BLC). (**A**) Thermal stability of BLC alone (0.2 μM) and in the presence of CLZ (10 μM). (**B**) Effect of 10 mM 2,2′-azobis(2-amidinopropane) dihydrochloride-induced oxidation of BLC (0.1 μM) alone and in the presence of increasing concentrations of CLZ (0–10 μM).

Thermal stability is a key determinant of protein structural integrity and can be influenced by specific ligand binding [41]. Shifts in melting temperature (T_m_) can signify stabilization or destabilization of the native enzyme structure. In our study, the thermal stability of BLC was assessed by monitoring changes in its intrinsic fluorescence as temperature increased, both in the absence and in the presence of a 50-fold molar excess of CLZ (Figure 3A). The T_m_ value for catalase alone, the temperature at which 50% of the enzyme is unfolded or inactivated, was determined to be 57.8 °C, in accordance with literature data [42]. In the presence of CLZ, an increase was observed, with the T_m_ rising to 59.4 °C. This result indicates that drug binding slightly stabilises the enzyme structure, making it more resistant to thermal denaturation. This result aligns with CD spectroscopy data, confirming that the tertiary structure of BLC is more rigid in the presence of CLZ. Additionally, an increase in the *β*-barrel motif and a decrease in the α-helix during drug binding may also contribute to increased thermal stability, as the *β*-barrel is a thermally more stable protein structure [43].

#### 2.2.3. Effect of Clozapine on Bovine Liver Catalase Stability in an Oxidative Environment

Complex formation generally increases enzyme stability in an oxidative environment by inducing a more compact, rigid, and protected structure that shields critical residues from ROS [44]. 2,2′-Azobis(2-amidinopropane) dihydrochloride (AAPH) decreases protein fluorescence primarily by inducing oxidative damage to amino acid residues, thereby decreasing the intrinsic fluorescence intensity. This process is driven by the production of peroxyl and alkoxyl radicals, which lead to protein unfolding and, at higher concentrations, structural aggregation [45].

The addition of AAPH to the BLC solution (Section 3.2) resulted in extensive chemical modifications to enzyme, as evidenced by a substantial reduction in its fluorescence over time (Figure 3B). This effect likely reflects chemical alterations to Trp residues, which are particularly susceptible to oxidative modification by AAPH [45]. With the addition of CLZ, a dose-dependent protective trend was observed (Figure 3B). At all concentrations, CLZ slowed the fluorescence decay, suggesting that this neuroleptic, at the used concentrations, provided protection and stabilisation of BLC structure under oxidative stress. This effect reflects CLZ’s in vitro antioxidative capacity [46,47] and its ability to protect critical catalase residues from radical-induced damage, as previously shown in studies of CLZ binding to fibrinogen and *α*2-macroglobulin [17,18].

These results are consistent with prior studies suggesting that CLZ, when administered at controlled concentrations, may exert in vivo antioxidant effects as well, e.g., [48]. The partial protective effect observed in this study supports CLZ’s potential to preserve BLC structure and activity under oxidative conditions, further illustrating its context-dependent redox properties. It could be relevant in its therapeutic use, in neurodegenerative and psychiatric conditions associated with prolonged oxidative stress [49].

#### 2.2.4. Effect of Complex Formation on Bovine Liver Catalase Activity

Catalase is a pivotal antioxidant enzyme vital for maintaining cellular redox homeostasis and preventing damage to DNA, proteins, and lipids in aerobic organisms, especially under high-stress conditions, metabolic disorders, or neurodegeneration [50]. Standard enzyme assay and zymography (Section 3.5) were used to investigate the effect of CLZ binding on BLC activity.

**Figure 4:**
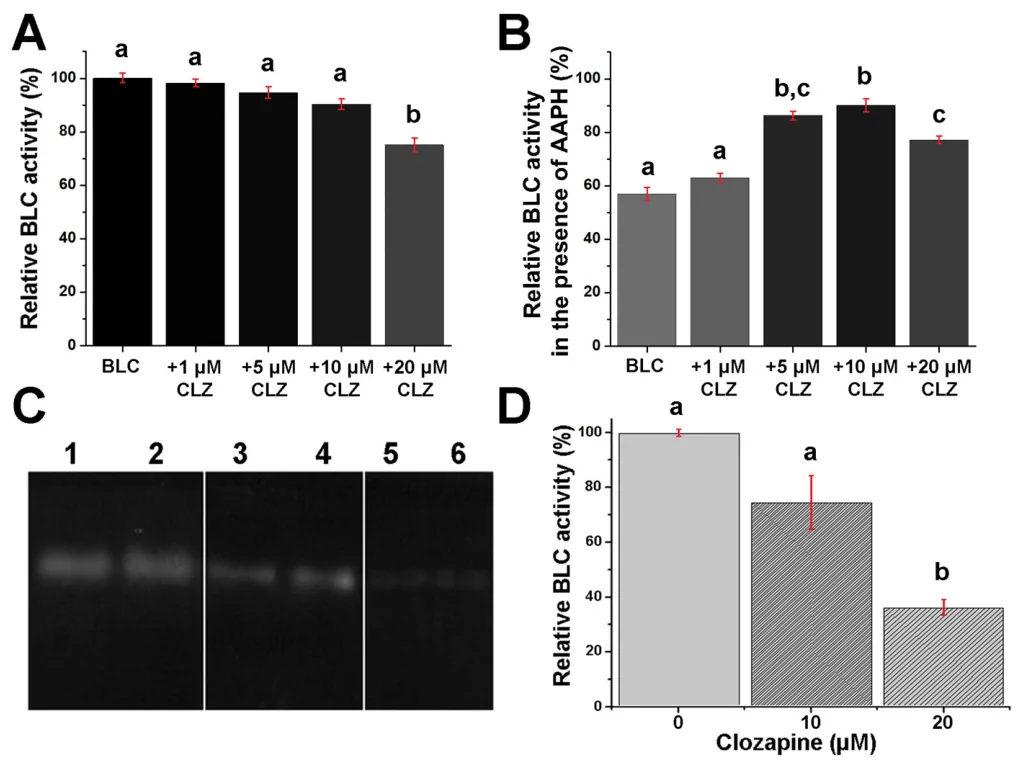
Effects of clozapine (CLZ) binding on bovine liver catalase (BLC) activity. (**A**) The effect of various concentrations of CLZ (0–20 μM) on BLC activity (0.1 μM) in 50 mM phosphate buffer, pH 7.2, at 37 °C. (**B**) Effect of free radical-induced oxidation (10 mM 2,2′-azobis(2-amidinopropane) dihydrochloride, AAPH) on BLC (0.1 μM) activity in the presence of increased concentrations of clozapine (0–10 μM). (**C**) Representative zymogram of BLC (500 ng), alone—lanes 1 and 2, in the presence of 10 μM CLZ—lanes 3 and 4, and 20 μM CLZ—lanes 5 and 6. (**D**) Densitometric quantification of BLC bands from (**C**). Error bars represent the standard deviation; n = 3 (**A**,**B**) or n = 2 (**D**). Different letters on histograms indicate statistical difference (*p* < 0.05) between the samples.

In the spectrophotometric assay, in which BLC (0.1 μM) was incubated with CLZ at 1 μM, 5 μM, 10 μM, and 20 μM, we observed a gradual decrease in enzyme activity with increasing drug concentration (Figure 4A). Specifically, BLC activity remained similar to control levels at lower CLZ concentrations. However, a significant reduction (*p* < 0.05) was observed at 20 μM, indicative of concentration-dependent BLC inhibition by CLZ. Zymographic analysis (Figure 4C) confirmed this trend, showing a progressive decline in BLC activity in the gel with increasing CLZ exposure. This method assessed BLC activity (500 ng) on a polyacrylamide gel in the presence of 10 μM and 100 μM CLZ. The untreated BLC showed the highest colorless band intensity against a stained background, indicating the highest enzyme activity (100%). Densitometry of activity bands showed that at 10 μM CLZ, enzyme activity was reduced to 74%, and it dropped further to 35.9% at 20 μM CLZ, confirming a dose-dependent inhibition of BLC by CLZ, as illustrated in Figure 4D.

To examine the effect of CLZ complexation on BLC catalytic activity under oxidative stress conditions, AAPH (10 mM) was added to the same mixture in phosphate buffer (Section 3.5). As expected, the activity of pure enzyme decreased substantially in the presence of AAPH, reflecting the enzyme’s vulnerability to oxidative damage by free radicals (Figure 4B). When CLZ was added at low concentrations (5 μM and 10 μM) along with AAPH, we observed almost complete preservation of BLC activity (cca. 90%) compared to the AAPH-only condition, confirming a protective antioxidant effect of this drug at lower doses. However, as the CLZ concentration increased to 20 μM, the observed inhibitory effect on CLZ binding was accompanied by a protective effect, resulting in a reduction in BLC activity to values similar to those of the appropriate control without AAPH (Figure 4B vs. Figure 4A).

These findings on changes in BLC activity upon CLZ binding in (patho)physiological settings align with previous studies reporting CLZ’s complex, dose-dependent, and sometimes opposite effects on cellular redox balance. At concentrations less than 15 μM, CLZ and its main metabolite (*N*-desmethylclozapine) may offer limited protection against oxidative stress through direct free-radical scavenging, particularly in the cytosolic compartment [48,51], in agreement with the results of this study. Based on preclinical studies, CLZ induced significant oxidative damage at concentrations of 25 μM and more [52,53]. This phenomenon is associated with bioactivation into reactive metabolites (e.g., nitrenium ions), increased ROS formation, reduced antioxidant defenses, and mitochondrial dysfunction [52]. Our results convincingly showed that CLZ inhibited BLC antioxidant activity at the similar (high) concentrations. This concentration-dependent interaction underscores the importance of careful dosing in CLZ therapy, as its pleiotropic effects on antioxidant enzymes, such as catalase, may disrupt redox balance under chronic or high-dose treatment conditions. However, the real, significant, harmful effects of CLZ on the primary antioxidative protection system in circulation seem unlikely. The therapeutic plasma concentration range for CLZ is generally much lower, typically 350–600 ng/mL, while levels above 1000 ng/mL (or 3.1 μM) have been linked to a perceived risk of developing full-blown toxicity [54].

### 2.3. Molecular Docking and Molecular Dynamics of Clozapine Binding to Bovine Liver Catalase

**Figure 5:**
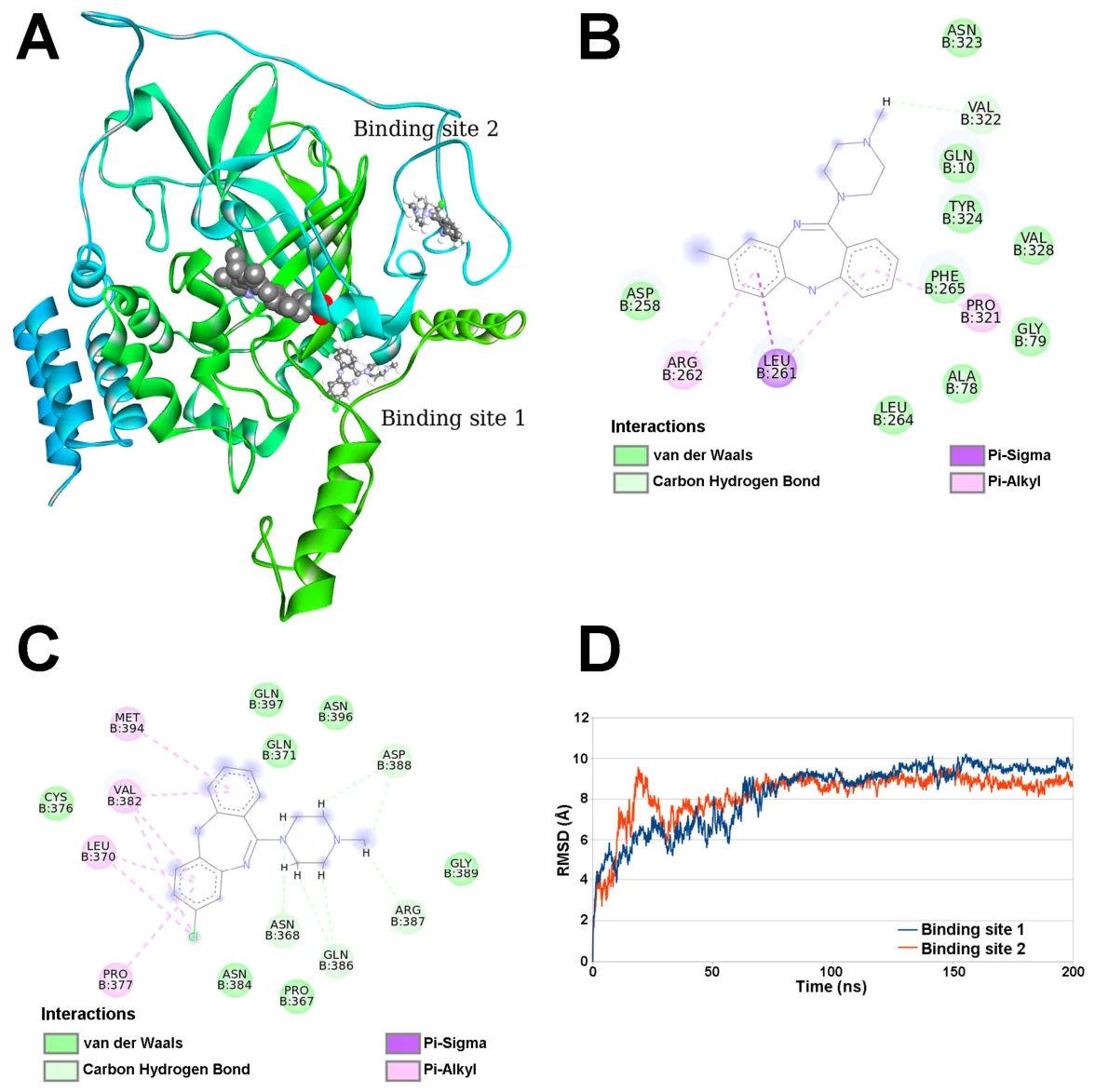
Molecular modeling results on the bovine liver catalase–clozapine (BLC–CLZ) complex. (**A**) Binding sites of CLZ *P* and *M* atropisomers in BLC with heme derived from molecular docking simulations; (**B**,**C**) 2D representations of clozapine *P* atropisomer interactions with BLC at binding sites 1 and 2, respectively, obtained through molecular docking; (**D**) Root Mean Square Deviation (RMSD) graph of molecular dynamics simulations at binding site 1 (blue) and binding site 2 (red).

Molecular docking and MD simulations are complementary in silico techniques commonly used to predict, analyze, and refine protein–drug interactions. Docking rapidly predicts binding poses and affinity, while MD simulations provide a time-dependent, flexible view of the complex to evaluate stability and refine binding mechanisms [55]. CLZ exists as distinct conformational isomers known as atropisomers (*P* or plus, and *M* or minus), arising from restricted rotation around the chiral axis of the molecule’s diazepine ring [56]. The docking study on the BLC monomer (Section 3.6) revealed two identical binding sites for both the *P* and *M* atropisomers of this drug (Figure 5), with the conformers showing similar affinities for both sites.

In the first binding site, both the *P* and *M* atropisomers of CLZ interact with residues positioned next to the *β*-barrel, between the helical domain and the threading arm of the BLC monomer (Figure 5A). This region of the enzyme structure is particularly interesting because it has previously been reported as a ligand-binding site, such as for resveratrol, suggesting it might play a role in ligand recognition and binding affinity. Although they appear to share a binding site, the binding constant of resveratrol to BLC was lower than that of CLZ (2.6 × 10^3^ M^−1^ at 37 °C), yet it still caused structural changes that reduced its catalytic function [57]. The *P* and *M* atropisomers showed similar binding scores of −7.8 kcal mol^−1^ and −7.7 kcal mol^−1^, respectively, for binding site 1, indicating that both CLZ isomers can effectively bind to this site. Regarding specific interactions within this binding site, CLZ is located near Tyr324 and interacts with Leu261, Arg262, Pro321, and Val322 (Figure 5B). Leu261 and Arg262 residues are located at secondary structural element *α*-helix 7, while Pro321 and Val322 are at an unorganized loop nearby [16]. Small ligand binding at this site may influence the enzyme’s stability and flexibility, regulating its overall conformational dynamics.

The second binding site, located more centrally within the only *β*-barrel structure and wrapping domain (Figure 5A), appears to be another significant ligand-recognition region for BLC. It has been associated with the binding of polyphenolic compounds such as quercetin and ellagic acid. Interestingly, quercetin (association constant of 10^4^ M^−1^), like CLZ, efficiently inhibited BLC in vitro [58], in contrast to nearly two-fold increases in enzymatic activity by ellagic acid binding (*K*_a_ of 17.6 × 10^7^ M^−1^ at 37 °C) [59]. Again, both *P*- and *M*-atropisomers of CLZ had similar binding scores of −7.8 kcal mol^−1^ and −7.5 kcal mol^−1^, respectively. This site involves interactions with residues such as Asn368, Leu370, Pro377, Val382, Arg387, Asp388, and Met394 (Figure 5C). The proximity of CLZ-binding residues to the wrapping domain suggests conformational changes in the adjacent *β*-barrel, which could influence overall enzyme stability and explain the obtained increase in T_m_ value (Section 2.2.2).

MD simulations confirmed that the BLC–CLZ complex maintained structural stability throughout the simulation. It was supported by relatively constant Root Mean Square Deviation (RMSD) values (plateauing) of the backbone atoms after an initial equilibration period (Figure 5D), indicating that the antipsychotic drug was stably anchored at both predicted binding sites on the BLC monomer.

Binding site 2 for CLZ is located away from the BLC active site, and it is implausible that it can influence the enzyme’s activity. On the other hand, while binding site 1 does not include any amino acid residues directly involved in catalytic activity of catalase [60], nor is it near to tightly bound NADPH inside the monomer unit, its position is in relative proximity to active site and at higher CLZ concentrations, at which this site is saturated more than 50% (estimation based of the obtained affinity constant), it may influence (reduce) the activity of BLC to some extent, as shown by our experimental results.

## 3. Materials and Methods

### 3.1. Materials

All chemicals used, including bovine liver catalase (Product No. C-9322; 2000–5000 units/mg protein), were purchased from Sigma (St. Louis, MO, USA). A stock solution of enzyme (20 μM) was prepared in 50 mM phosphate buffer, pH 7.2, dialyzed against the same buffer, and centrifuged for 1 min at 12,000× *g* before use. The concentration of BLC was determined by measuring its absorbance at 276 nm using a 1% extinction coefficient of 12.9 [61]. Clozapine was provided by Remedica Ltd. (Limassol, Cyprus). A stock solution of the drug (24 mM) was prepared in HPLC-grade methanol. In all experiments, the ultrapure (Milli-Q^®^) water (Merck Millipore, Darmstadt, Germany) was used to prepare all solutions, with the final methanol concentration not exceeding 1%.

### 3.2. Spectrofluorimetric Measurements

All fluorimetric measurements were performed on a FluoroMax^®^-4 (Horiba Scientific, Kyoto, Japan) spectrofluorometer equipped with a Peltier heating/cooling system.

Standard intrinsic protein fluorescence quenching assays were employed to characterize the binding affinity and thermodynamic parameters of CLZ with BLC. Measurements were performed in triplicate at physiologically relevant temperatures (25 °C, 30 °C, and 37 °C) to assess temperature-dependent binding dynamics. The fluorescence emission spectra of native BLC (0.1 μM) in the absence and presence of increasing concentrations of CLZ (0.1–1 μM) were recorded with an excitation wavelength (λ_ex_) set at 280 nm. The emission wavelength (λ_em_) ranged from 290 to 440 nm, with two accumulations and slits set at 5 nm; spectra were recorded after a 2-min BLC incubation at each drug concentration. The obtained average emission spectra were corrected by subtracting emission spectra from CLZ alone.

The following equation was used to correct the values of emission maxima at 340 nm for the inner filter effect [62]:

F_c_ = F_0_ × 10^(Aem × dem + Aex × dex)/2^ (1)

where F_c_ is the corrected fluorescence, F_0_ is the measured fluorescence, while A_ex_ and A_em_ correspond to absorbances at a wavelength of excitation (280 nm) and emission peak (340 nm) of clozapine at a particular concentration. Optical path lengths for both excitation (d_ex_) and emission directions (d_em_) were 1 cm.

The association (binding) constant (*K*_a_) was calculated using the following equation [63]:

(2) $$\frac{1}{r}\;=\;\frac{1}{n\;\times \;K_{a}}\times \frac{1}{\left[L_{f}\right]}+\frac{1}{n}$$

where r represents moles of bound ligand per mol of protein, [L_f_] is the molar concentration of free ligand, and n is the number of binding sites on the protein. Concentration of [L_f_] was calculated from fluorescence spectra using the following equation [64]:

(3) $$\left[L_{f}\right]\;=\;\left[L_{t}\right]\;-\;\frac{\left(F_{0}-F_{c}\right)}{F_{0}}\;\times \;[P_{t}]$$

where [L_t_] is the total ligand (CLZ) concentration, [P_t_] is the total protein concentration, [L_f_] is the concentration of free ligand, F_0_ is the fluorescence intensity of BLC without ligand, and F_c_ is the fluorescence intensity in the presence of ligand. The concentration of bound ligand was calculated by subtracting [L_f_] from [L_t_].

For determination of the type of quenching, which may be dynamic (collisional) or static (due to the complex formation), or dual, the Stern–Volmer (SV) plot was obtained using the equation [62]:

F_0_/F = 1 + *k*_q_ *τ*_0_ [Q] = 1 + *K*_sv_ [Q] (4)

where F_0_ and F are the emission signals of BLC in the absence and the presence of CLZ at 340 nm, *k*_q_ is the bimolecular quenching rate constant, *τ*_0_ is the average lifetime of the protein without quencher, for catalase 5 × 10^−9^ s [28], [Q] is the quencher (CLZ) concentration, and *K*_sv_ is the SV quenching constant.

To determine the thermodynamic parameters of protein-drug binding, the van’t Hoff equation was applied:

ln*K*_a_ = −∆*H*/RT + ∆*S*/R (5)

where *K*_a_ is the association constant at the corresponding temperature, R is the universal gas constant (8.314 J mol^−1^ K^−1^), and T is the absolute temperature (K). There is a linear relationship between ln*K*_a_ and 1/T, from which the enthalpy change Δ*H* can be obtained from the slope of the plot and Δ*S* from the y-intercept. The free energy change (Δ*G*) was calculated using the Gibbs-Helmholtz equation:

∆*G* = ∆*H* − T∆*S* (6)

Synchronous fluorescence spectra of 0.1 µM BLC alone and in the presence of rising concentration of CLZ, from 0.1 to 1 µM, were collected at 37 °C using the scanning interval (Δλ = λ_em_ − λ_ex_) set at 15 nm to analyze the conformational changes and microenvironmental variations around Tyr residues and at 60 nm to assess alterations related to Trp residues upon drug binding.

The thermal stability of BLC alone (0.2 μM) or in the presence of CLZ (10 μM) was investigated in the temperature range of 37 to 81 °C by quantifying the reduction in enzyme intrinsic fluorescence due to protein unfolding. The temperature was increased at 1 °C per minute, with a 1-min equilibration time and 5 nm slits. The melting temperature of BLC is defined as the inflection point of the sigmoidal curve obtained by plotting F_330 nm_/F_350nm_ versus temperature [65].

The reduction in intrinsic fluorescence of 0.1 μM BLC, alone or in the presence of 1 μM, 5 μM, and 10 μM CLZ, was measured in duplicate in the free radical-induced protein oxidation test. To induce oxidative stress and evaluate the enzyme’s vulnerability to oxidation, BLC samples were exposed to 10 mM AAPH—a water-soluble, heat-labile azo compound that generates free radicals at physiological temperatures via decomposition. The resulting decrease in intrinsic protein fluorescence indicated oxidative modification of aromatic residues, particularly Trp [66]. Changes in the emission spectra of BLC were monitored upon excitation at 280 nm for 30 min at 37 °C, using a 5 nm slits width.

### 3.3. UV Spectroscopic Measurements

Absorption spectra of the sample were obtained with a UV-1800 spectrophotometer (Shimadzu, Kyoto, Japan) in a 1 cm cuvette at 37 °C. The absorbance of BLC solution alone (4.8 μM) and in the presence of 4.8 μM, 19.2 μM, and 48 μM CLZ was recorded between 260 and 320 nm, with a bandwidth of 1 nm and a data interval of 0.1 nm, at a scanning speed of 30 nm per minute. Enzyme spectra were baseline-corrected by subtracting emission signals from the drug alone. The fourth-derivative spectra were calculated in OriginPro 8.5 using the Savitzky–Golay smoothing algorithm with a 100-point window.

### 3.4. Circular Dichroism Spectroscopy Measurements

The influence of clozapine binding on the structure of the BLC macromolecule was investigated using circular dichroism (CD) spectroscopy on a JASCO CD J-815 spectropolarimeter (JASCO Corporation, Hachioji, Japan) with a Peltier temperature controller set to 37 °C. The recordings were made using a quartz cuvette in the far-UV range (185 to 260 nm) for enzyme alone (12 μM) and in the presence of 12 μM and 120 μM drug with 0.5 mm path length, and the near-UV range (260 to 320 nm), for catalase alone (8 μM) and in the presence of 8 μM, 32 μM, and 80 μM CLZ with an optical path length of 10 mm. All CD spectra, obtained with three accumulations and a scanning speed of 50 nm per minute, were baseline-corrected by subtracting an appropriate blank (phosphate buffer) spectrum of the drug solutions. The CD-pro program, utilizing the CONTIN algorithm (https://www.bmb.colostate.edu/cdpro/), accessed on 19 April 2025, was employed to assess changes in the percentages of secondary and unordered structural elements of BLC in the absence and presence of increasing concentrations of CLZ.

### 3.5. Bovine Liver Catalase Activity Measurements

The activity of BLC was determined using a standard spectrophotometric kinetic assay, monitoring a decrease in hydrogen peroxide absorbance at 240 nm due to its decomposition by this enzyme on a UV-1800 spectrophotometer (Shimadzu, Japan) in a 1.0 cm cuvette at 37 °C for 3 min. The rate of decomposition (ΔA/minute) is converted to molar units using the extinction coefficient for H_2_O_2_ of 43.6 M^−1^ cm^−1^ at 240 nm [61]. BLC (0.1 μM in 50 mM phosphate buffer, pH 7.2) was incubated with 1 μM, 5 μM, 10 μM, and 20 μM CLZ for 5 min. Then, 40 μL of the resulting solution was added to the reaction mixture containing 12 mM H_2_O_2_ in the same buffer, for a total reaction volume of 2 mL. To investigate whether the presence of CLZ protects BLC activity under oxidative conditions, the reaction mixtures described above were incubated with 10 mM AAPH for 30 min in a separate experiment before activity measurement. One unit of BLC activity is defined as the amount of enzyme that decomposes 1 µM of H_2_O_2_ per min. The activity of BLC alone was set to 100%, and the activity results are normalized to this value.

BLC activity was additionally assessed using polyacrylamide gel electrophoresis (PAGE)-based zymography, following established protocols to visualize enzyme activity in situ [67]. Each lane of a 10% resolving gel was loaded with 500 ng of BLC. After electrophoresis, the gel was cut into 3 pieces, each washed 3 times with distilled water (10 min each), then incubated in 50 mM phosphate buffer, pH 7.2, alone or in the presence of 10 μM or 20 μM CLZ. After 30 min, the gels were incubated in 0.04 M H_2_O_2_ for 10 min in the same buffer. Then, the gels were washed with distilled water for 5 min and subsequently incubated in a freshly prepared solution containing 2% ferric chloride and 2% potassium ferricyanide, which produced a dark green background upon reaction with residual peroxide. Active catalase appeared as distinct white bands, indicating localized hydrogen peroxide degradation. Band intensities were quantified using ImageJ software (v2.0.0-rc-15), providing semi-quantitative measurements of enzyme activity across treatment conditions. The intensity of bands obtained for BLC alone was set to 100%, and the other band intensities were normalized accordingly.

### 3.6. Molecular Docking Calculation

The BLC crystal structure (PDB ID: 1TGU) was downloaded from the Protein Data Bank (https://www.rcsb.org/). Since all four catalase chains are identical, chain B was chosen for the molecular docking simulation. All water molecules from the selected chain were deleted from the structure, and the H++ program 3.0 [68] was used to estimate the protonation state of each titratable amino acid residue. To correct possible unfavorable contacts in the crystal structure, the chosen chain was optimized for 3000 steps using CHARMM (version c35b1) [69]. Like many other compounds with a 1,4-benzodiazepine group [70], CLZ occurs in the form of two atropisomers (*P* and *M*) [56], which are also enantiomers. To determine the structures of both atropisomers of the CLZ molecule, the Cambridge Structural Database (CSD) [71] was scanned. The structures with CSD Refcode HIHHOY01 and DEHBUP (representing *P* and *M* atropisomers, respectively) were selected [72]. The structure of the drug ligand in both forms was optimized as previously described [18]. Finally, optimized enzyme and ligand structures were arranged for docking with the AutoDockTools program, version 1.5.6. Sep_17_14 [73]. The molecular docking simulation was performed using the AutoDock Vina program [74]. The ligand single bond between 1,4-diazepine and piperazine rings was set to be rotational, while all protein residues were kept rigid. A grid box with 28 × 28 × 28 Å accommodated the ligand to move freely during a blind docking simulation. To cover the entire volume of protein, a grid box was shifted over the rectangular matrix containing the protein, with points spaced 8 Å apart. A total of 1260 docking runs, with the exhaustiveness parameter set to 100, were performed for BLC (with heme), and this was done twice for both atropisomers of the CLZ molecule. Docking poses were clustered and ranked by binding score. The conformations with the lowest binding scores (highest affinity) within the predominant clusters were selected as the most probable binding modes.

### 3.7. Molecular Dynamics Calculation

Molecular dynamics simulations further investigated the stability of the BLC–CLZ complex identified by docking. The Amber software (version 24) with GPU support [74] was employed for MDS. The AmberTools suite (version 23) [75] was used to prepare the enzyme and ligand and to analyze the results. The ff19SB protein force field was selected for BLC, while the General Amber Force Field (gaff2) was utilized for CLZ. The metal center parameters were generated using MCPB.py from the AmberTools 23 suite [76]. The BLC-CLZ complex was solvated in a truncated octahedral box (dimensions 114.021 × 114.021 × 114.021 Å, total volume 760,851.0 Å^3^) with the OPC 4-point water model [77], with periodic boundary conditions set.

The system was initially minimized for 2000 steps using the Newton-Raphson algorithm, followed by a progressive increase in temperature from 100 K to 298 K over 1 ns, with constant volume maintained. The system was equilibrated for 6 ns with a time step of 1 fs in the constant-temperature, constant-pressure ensemble (NPT), gradually reducing the constraints on protein and peptide atoms to 0.1 kcal/mol Å^2^. A final equilibration of 5 ns in the NPT ensemble was conducted without constraints, using a 1 fs time step. For a 200 ns production run, the NPT ensemble was employed, with a Langevin thermostat and a Berendsen barostat, using a 2 fs time step. The SHAKE algorithm was used to constrain hydrogen atoms. Molecular dynamics trajectory analysis was performed using the VMD program [78].

### 3.8. Statistical Analysis

The results for binding affinity, SV constant, bimolecular quenching rate constant, and catalase activity are presented as the mean ± S.D (Standard Deviation). Where applicable, data were analyzed using one-way ANOVA with Tukey’s multiple comparison test at a significance level of 0.05.

## 4. Conclusions

This study comprehensively characterized the in vitro interaction between CLZ, the “gold standard” medication for treatment-resistant schizophrenia, and BLC, an exceptionally efficient ubiquitous antioxidant enzyme. It provided insights into the binding affinity of CLZ, structural remodeling, thermal stability, oxidative protection, and BLC activity. Fluorescence quenching analyses revealed a moderate binding affinity, in the order of 10^5^ M^−1^, with Stern–Volmer constants supporting a static quenching mechanism. Negative ΔG values confirmed spontaneous binding, and enthalpy–entropy analysis indicated that the hydrophobic effect was the dominant stabilizing force, as observed in prior drug–catalase interaction studies. CD spectroscopy indicates increased structural rigidity of BLC upon CLZ binding, which was confirmed by the enzyme’s higher thermal stability in the presence of the drug. Bovine liver catalase activity assays demonstrated dose-dependent inhibition, highlighting a protective effect at lower (physiologically achievable) CLZ concentrations but inhibition at higher (toxic) doses. Molecular docking and molecular dynamics analysis predicted two specific binding sites for CLZ within the BLC subunit, similar to those of other lipophilic bioactive ligands that modulate the catalytic activity of this enzyme. Overall, the dualistic, concentration-dependent impact of CLZ on BLC could provide biochemical insight into its complex beneficial and adverse pleiotropic effects. Further studies are essential to translate these findings to clinical contexts.

## Figures and Tables

**Table 1:** Binding parameters of the bovine liver catalase–clozapine complex at 25 °C, 30 °C, and 37 °C in 50 mM phosphate buffer pH 7.2.

T (°K)	*K*_a_ × 10^5^ (M^−1^)	*K*_sv_ × 10^5^ (M^−1^)	*k*_q_ × 10^13^ (M^−1^ s^−1^)	Δ*G* (kJ mol^−1^)	Δ*H* (kJ mol^−1^)	ΔS (J mol^−1^ K^−1^)
298	1.37 ± 0.03	2.18 ± 0.07	4.36 ± 0.05	−29.31 ± 0.023		
303	1.27 ± 0.01	2.04 ± 0.07	4.08 ± 0.04	−29.61 ± 0.022	−17.8 ± 0.12	38.8 ± 0.48
310	1.04 ± 0.02	1.73 ± 0.03	3.46 ± 0.03	−29.79 ± 0.026		

Data are presented as means ± standard deviation from two independent, duplicate experiments. *K*_a_, binding (association) constant; *K*_sv_, Stern–Volmer quenching constant; *k*_q_, quenching rate constant.

**Table 2:** Content of secondary structure elements of bovine liver catalase (BLC) with and without bound clozapine (CLZ) at 37 °C.

Molar Ratio BLC to CLZ	*α*-Helix (%)	*β*-Sheet (%)	*β*-Turn (%)	Random Coil (%)
1:0	23.7 ± 0.60	17.5 ± 0.51	25.5 ± 0.61	33.3 ± 0.56
1:1	22.9 ± 0.71	20.4 ± 0.70	24.9 ± 0.72	31.8 ± 0.67
1:10	21.7 ± 0.54	21.2 ± 0.59	24.7 ± 0.60	32.4 ± 0.64

Data are presented as means ± standard deviation from two independent experiments.

## Data Availability

The original contributions presented in this study are included in the article. Further inquiries can be directed to the corresponding authors.

## References

[1] Fanshawe J.B., Kabir T., Lennox B.R. (2025). Clozapine: Old drugs require efforts to improve patient experience and access. Lancet.

[2] Gerlach J., Lublin H., Peacock L. (1996). Extrapyramidal symptoms during long-term treatment with antipsychotics: Special focus on clozapine and D1 and D2 dopamine antagonists. Neuropsychopharmacology.

[3] Khokhar J.Y., Henricks A.M., Sullivan E.D.K., Green A.I. (2018). Unique Effects of Clozapine: A Pharmacological Perspective. Adv. Pharmacol..

[4] Gurrera R.J., Gearin P.F., Love J., Li K.J., Xu A., Donaghey F.H., Gerace M.R. (2022). Recognition and management of clozapine adverse effects: A systematic review and qualitative synthesis. Acta Psychiatr. Scand..

[5] Kar N., Barreto S., Chandavarkar R. (2016). Clozapine Monitoring in Clinical Practice: Beyond the Mandatory Requirement. Clin. Psychopharmacol. Neurosci..

[6] Correll C.U., Agid O., Crespo-Facorro B., de Bartolomeis A., Fagiolini A., Seppälä N., Howes O.D. (2022). A Guideline and Checklist for Initiating and Managing Clozapine Treatment in Patients with Treatment-Resistant Schizophrenia. CNS Drugs.

[7] Murray A.J., Rogers J.C., Katshu M.Z.U.H., Liddle P.F., Upthegrove R. (2021). Oxidative Stress and the Pathophysiology and Symptom Profile of Schizophrenia Spectrum Disorders. Front. Psychiatry.

[8] Li M., Bai Y., Wang Y., Xing H., Zhang Y., Ding W., Ye H., Ma L., Wang W., Bao T. (2025). A Review of Clinical Advances and Challenges in Clozapine-Induced Myocarditis. Neuropsychiatr. Dis. Treat..

[9] Fehsel K., Loeffler S., Krieger K., Henning U., Agelink M., Kolb-Bachofen V., Klimke A. (2005). Clozapine induces oxidative stress and proapoptotic gene expression in neutrophils of schizophrenic patients. J. Clin. Psychopharmacol..

[10] Torii-Goto A., Yoshimi A., Tashiro Y., Ukigai M., Matsumoto A., Ozaki N., Noda Y. (2022). A Reactive Metabolite of Clozapine Induces Hematopoietic Toxicity in HL-60 Cells Undergoing Granulocytic Differentiation through Its Effect on Glutathione Metabolism. Biol. Pharm. Bull..

[11] Magliaro B.C., Saldanha C.J. (2009). Clozapine protects PC-12 cells from death due to oxidative stress induced by hydrogen peroxide via a cell-type specific mechanism involving inhibition of extracellular signal-regulated kinase phosphorylation. Brain Res..

[12] Lundberg M., Curbo S., Bohman H., Agartz I., Ögren S.O., Patrone C., Mansouri S. (2020). Clozapine protects adult neural stem cells from ketamine-induced cell death in correlation with decreased apoptosis and autophagy. Biosci. Rep..

[13] Nandi A., Yan L.J., Jana C.K., Das N. (2019). Role of Catalase in Oxidative Stress- and Age-Associated Degenerative Diseases. Oxid. Med. Cell. Longev..

[14] Zámocký M., Koller F. (1999). Understanding the structure and function of catalases: Clues from molecular evolution and in vitro mutagenesis. Prog. Biophys. Mol. Biol..

[15] Anwar S., Alrumaihi F., Sarwar T., Babiker A.Y., Khan A.A., Prabhu S.V., Rahmani A.H. (2024). Exploring Therapeutic Potential of Catalase: Strategies in Disease Prevention and Management. Biomolecules.

[16] Murthy M.R., Reid T.J., Sicignano A., Tanaka N., Rossmann M.G. (1981). Structure of beef liver catalase. J. Mol. Biol..

[17] Gligorijević N., Vasović T., Lević S., Miljević Č., Nedić O., Nikolić M. (2020). Atypical antipsychotic clozapine binds fibrinogen and affects fibrin formation. Int. J. Biol. Macromol..

[18] Šunderić M., Vasović T., Milčić M., Miljević Č., Nedić O., Nikolić M.R., Gligorijević N. (2021). Antipsychotic clozapine binding to alpha-2-macroglobulin protects interacting partners against oxidation and preserves the anti-proteinase activity of the protein. Int. J. Biol. Macromol..

[19] Lowe P.N., Vaughan C.K., Daviter T., Williams M., Daviter T. (2013). Measurement of Protein–Ligand Complex Formation. Protein-Ligand Interactions.

[20] Bakar K.A., Feroz S.R. (2019). A critical view on the analysis of fluorescence quenching data for determining ligand-protein binding affinity. Spectrochim. Acta A Mol. Biomol. Spectrosc..

[21] Minic S.L., Milcic M., Stanic-Vucinic D., Radibratovic M., Sotiroudis T.G., Nikolic M.R., Ćirković Velickovic T. (2015). Phycocyanobilin, a bioactive tetrapyrrolic compound of blue-green alga Spirulina, binds with high affinity and competes with bilirubin for binding on human serum albumin. RSC Adv..

[22] Cao Z., Liu R., Yang B. (2013). Potential toxicity of sarafloxacin to catalase: Spectroscopic, ITC and molecular docking descriptions. Spectrochim. Acta A Mol. Biomol. Spectrosc..

[23] Teng Y., Zou L., Huang M., Zong W. (2014). Molecular interaction of 2-mercaptobenzimidazole with catalase reveals a potentially toxic mechanism of the inhibitor. J. Photochem. Photobiol. B.

[24] Albani J.R., Albani J.R. (2004). Chapter 4—Fluorescence Quenching. Structure and Dynamics of Macromolecules: Absorption and Fluorescence Studies.

[25] Stroppolo M.E., Falconi M., Caccuri A.M., Desideri A. (2001). Superefficient enzymes. Cell. Mol. Life Sci..

[26] Yekta R., Dehghan G., Rashtbari S., Sheibani N., Moosavi-Movahedi A.A. (2017). Activation of catalase by pioglitazone: Multiple spectroscopic methods combined with molecular docking studies. J. Mol. Recognit..

[27] Zou L., Mi C., Yu H., Gu W., Teng Y. (2017). Characterization of the interaction between triclosan and catalase. RSC Adv..

[28] Yang Y., Li D. (2016). Investigation on the interaction between isorhamnetin and bovine liver catalase by spectroscopic techniques under different pH conditions. Luminescence.

[29] Zaman M., Nusrat S., Zakariya S.M., Khan M.V., Ajmal M.R., Khan R.H. (2017). Elucidating the interaction of clofazimine with bovine liver catalase: A comprehensive spectroscopic and molecular docking approach. J. Mol. Recognit..

[30] Wu Y. (2007). Study on the interaction between salicylic acid and catalase by spectroscopic methods. J. Pharm. Biomed. Anal..

[31] Zhou L., Zhou H., Xiao H., Zhang Z., Xiong Z., Tuo X., Guo H. (2022). Elucidation on inhibition and binding mechanism of bovine liver catalase by nifedipine: Multispectroscopic analysis and computer simulation methods. Luminescence.

[32] Wang R., Zhang L., Wang R., Dou H., Li H., Wang Y., Pu J., Wang R. (2013). Spectroscopic study on the interaction of catalase with bifendate and analogs. Spectrochim. Acta A Mol. Biomol. Spectrosc..

[33] Ferenczy G.G., Keserű G.M., Luque J., Barril X. (2012). Physico-Chemical and Computational Approaches to Drug Discovery.

[34] Du X., Li Y., Xia Y.L., Ai S.M., Liang J., Sang P., Ji X.L., Liu S.Q. (2016). Insights into Protein-Ligand Interactions: Mechanisms, Models, and Methods. Int. J. Mol. Sci..

[35] Lange R., Frank J., Saldana J.-L., Balny C. (1996). Fourth derivative UV-spectroscopy of proteins under high pressure: I. Factors affecting the fourth derivative spectrum of the aromatic amino acids. Eur. Biophys. J..

[36] Kolakowski P., Dumay E., Cheftel J.-C. (2001). Effects of high pressure and low temperature on β-lactoglobulin unfolding and aggregation. Food Hydrocoll..

[37] Pal S., Dey S.K., Saha C. (2014). Inhibition of catalase by tea catechins in free and cellular state: A biophysical approach. PLoS ONE.

[38] Sreerama N., Woody R.W. (2000). Estimation of protein secondary structure from circular dichroism spectra: Comparison of CONTIN, SELCON, and CDSSTR methods with an expanded reference set. Anal. Biochem..

[39] Bucher D., Grant B.J., McCammon J.A. (2011). Induced fit or conformational selection? The role of the semi-closed state in the maltose binding protein. Biochemistry.

[40] Kelly S.M., Price N.C. (2000). The use of circular dichroism in the investigation of protein structure and function. Curr. Protein Pept. Sci..

[41] Waldron T.T., Murphy K.P. (2003). Stabilization of proteins by ligand binding: Application to drug screening and determination of unfolding energetics. Biochemistry.

[42] Feinstein R.N., Sacher G.A., Howard J.B., Braun J.T. (1967). Comparative heat stability of blood catalase. Arch. Biochem. Biophys..

[43] Zhang X.C., Han L. (2016). How does a β-barrel integral membrane protein insert into the membrane?. Protein Cell.

[44] Imlay J.A., Sethu R., Rohaun S.K. (2019). Evolutionary adaptations that enable enzymes to tolerate oxidative stress. Free Radic. Biol. Med..

[45] Fuentes-Lemus E., Dorta E., Escobar E., Aspée A., Pino E., Abasq M.L., Speisky H., Silva E., Lissi E., Davies M.J. (2016). Oxidation of free, peptide and protein tryptophan residues mediated by AAPH-derived free radicals: Role of alkoxyl and peroxyl radicals. RSC Adv..

[46] Dalla Libera A., Scutari G., Boscolo R., Rigobello M.P., Bindoli A. (1998). Antioxidant properties of clozapine and related neuroleptics. Free Radic. Res..

[47] Brinholi F.F., Farias C.C., Bonifácio K.L., Higachi L., Casagrande R., Moreira E.G., Barbosa D.S. (2016). Clozapine and olanzapine are better antioxidants than haloperidol, quetiapine, risperidone and ziprasidone in in vitro models. Biomed. Pharmacother..

[48] Zhao Q.L., Ito H., Kondo T., Uehara T., Ikeda M., Abe H., Saitoh J.I., Noguchi K., Suzuki M., Kurachi M. (2019). Antipsychotic drugs scavenge radiation-induced hydroxyl radicals and intracellular ROS formation, and protect apoptosis in human lymphoma U937 cells. Free Radic. Res..

[49] Chen X., Guo C., Kong J. (2012). Oxidative stress in neurodegenerative diseases. Neural. Regen. Res..

[50] Alhumaydhi F.A., Younus H., Khan M.A. (2025). Catalase Functions and Glycation: Their Central Roles in Oxidative Stress, Metabolic Disorders, and Neurodegeneration. Catalysts.

[51] Caruso G., Grasso M., Fidilio A., Tascedda F., Drago F., Caraci F. (2020). Antioxidant Properties of Second-Generation Antipsychotics: Focus on Microglia. Pharmaceuticals.

[52] Bakhshii S., Khezri S., Ahangari R., Jahedsani A., Salimi A. (2021). Protection of clozapine-induced oxidative stress and mitochondrial dysfunction by kaempferol in rat cardiomyocytes. Drug Dev. Res..

[53] Wang L., Chen Q., Ma R., Zhang B., Yang P., Cao T., Jiao S., Chen H., Lin C., Cai H. (2023). Insight into mitochondrial dysfunction mediated by clozapine-induced inhibition of PGRMC1 in PC12 cells. Toxicology.

[54] Skokou M., Karavia E.A., Drakou Z., Konstantinopoulou V., Kavakioti C.A., Gourzis P., Kypreos K.E., Andreopoulou O. (2022). Adverse Drug Reactions in Relation to Clozapine Plasma Levels: A Systematic Review. Pharmaceuticals.

[55] Santos L.H.S., Ferreira R.S., Caffarena E.R. (2019). Integrating Molecular Docking and Molecular Dynamics Simulations. Methods Mol. Biol..

[56] Rupard J.H., de Paulis T., Janowsky A., Smith H.E. (1989). Sterically hindered 5,11-dicarbo analogues of clozapine as potential chiral antipsychotic agents. J. Med. Chem..

[57] Rashtbari S., Dehghan G., Yekta R., Jouyban A., Iranshahi M. (2017). Effects of Resveratrol on the Structure and Catalytic Function of Bovine Liver Catalase (BLC): Spectroscopic and Theoretical Studies. Adv. Pharm. Bull..

[58] Rashtbari S., Dehghan G., Yekta R., Jouyban A. (2017). Investigation of the binding mechanism and inhibition of bovine liver catalase by quercetin: Multi-spectroscopic and computational study. Bioimpacts.

[59] Rashtbari S., Khataee S., Iranshahi M., Moosavi-Movahedi A.A., Hosseinzadeh G., Dehghan G. (2020). Experimental investigation and molecular dynamics simulation of the binding of ellagic acid to bovine liver catalase: Activation study and interaction mechanism. Int. J. Biol. Macromol..

[60] Sugadev R., Ponnuswamy M.N., Sekar K. (2011). Structural analysis of NADPH depleted bovine liver catalase and its inhibitor complexes. Int. J. Biochem. Mol. Biol..

[61] Herskovits T.T. (1969). Solvent perturbation studies of heme proteins and other colored proteins. Arch. Biochem. Biophys..

[62] Lakowicz J.R. (2004). Principles of Fluorescence Spectroscopy.

[63] Klotz I.M., Hunston D.L. (1971). Properties of graphical representations of multiple classes of binding sites. Biochemistry.

[64] Bi S., Ding L., Tian Y., Song D., Zhou X., Liu X., Zhang H. (2004). Investigation of the interaction between flavonoids and human serum albumin. J. Mol. Struct..

[65] Gligorijević N., Minić S., Radibratović M., Papadimitriou V., Nedić O., Sotiroudis T.G., Nikolić M.R. (2021). Nutraceutical phycocyanobilin binding to catalase protects the pigment from oxidation without affecting catalytic activity. Spectrochim. Acta A Mol. Biomol. Spectrosc..

[66] Werber J., Wang Y.J., Milligan M., Li X., Ji J.A. (2011). Analysis of 2,2′-azobis (2-amidinopropane) dihydrochloride degradation and hydrolysis in aqueous solutions. J. Pharm. Sci..

[67] Gamero-Sandemetrio E., Gómez-Pastor R., Matallana E. (2013). Zymogram profiling of superoxide dismutase and catalase activities allows Saccharomyces and non-Saccharomyces species differentiation and correlates to their fermentation performance. Appl. Microbiol. Biotechnol..

[68] Gordon J.C., Myers J.B., Folta T., Shoja V., Heath L.S., Onufriev A. (2005). H++: A server for estimating pKas and adding missing hydrogens to macromolecules. Nucleic Acids Res..

[69] MacKerell A.D., Bashford D., Bellott M., Dunbrack R.L., Evanseck J.D., Field M.J., Fischer S., Gao J., Guo H., Ha S. (1998). All-atom empirical potential for molecular modeling and dynamics studies of proteins. J. Phys. Chem. B..

[70] Meanwell N.A., Walker M.A., Katritzky A.R., Ramsden C.A., Scriven E.F.V., Taylor R.J.K. (2008). 13.06–1,4-Diazepines. Comprehensive Heterocyclic Chemistry III.

[71] Groom C.R., Bruno I.J., Lightfoot M.P., Ward S.C. (2016). The Cambridge Structural Database. Acta Crystallogr. B Struct. Sci. Cryst. Eng. Mater..

[72] Verma V., Bannigan P., Lusi M., Crowley C.M., Hudson S., Hodnetta B.K., Davern P. (2018). The heterogeneous crystallization of a novel solvate of clozapine base in the presence of excipients. CrystEngComm.

[73] Morris G.M., Huey R., Lindstrom W., Sanner M.F., Belew R.K., Goodsell D.S., Olson A.J. (2009). AutoDock4 and AutoDockTools4: Automated docking with selective receptor flexibility. J. Comput. Chem..

[74] Trott O., Olson A.J. (2010). AutoDock Vina: Improving the speed and accuracy of docking with a new scoring function, efficient optimization, and multithreading. J. Comput. Chem..

[75] Case D.A., Aktulga H.M., Belfon K., Cerutti D.S., Cisneros G.A., Cruzeiro V.W.D., Forouzesh N., Giese T.J., Götz A.W., Gohlke H. (2023). AmberTools. J. Chem. Inf. Model..

[76] Li P., Merz K.M. (2016). MCPB.py: A Python Based Metal Center Parameter Builder. J. Chem. Inf. Model..

[77] Izadi S., Anandakrishnan R., Onufriev A.V. (2014). Building Water Models: A Different Approach. J. Phys. Chem. Lett..

[78] Humphrey W., Dalke A., Schulten K. (1996). VMD: Visual molecular dynamics. J. Mol. Graph..

