# Correction: Vasović et al. Catalase Specifically Binds Antipsychotic Clozapine: Experimental and In Silico Insights into Interactions, Complex Stability, and Dose-Dependent Enzyme Activity Modulation. *Molecules* 2026, *31*, 1294

**DOI:** 10.3390/molecules31213958

**Published:** 2026-11-03

**Authors:** Tamara Vasović, Milica Radibratović, Dušan Spasić, Simeon Minić, Čedo Miljević, Nikola Gligorijević, Milan Nikolić

**Affiliations:** 1 Department of Biochemistry, Faculty of Chemistry, University of Belgrade, 11000 Belgrade, Serbia; tvasovic@chem.bg.ac.rs (T.V.); sminic@chem.bg.ac.rs (S.M.); 2 Department of Chemistry, Institute for Chemistry, Technology and Metallurgy, National Institute of the Republic of Serbia, University of Belgrade, 11000 Belgrade, Serbia; milica.radibratovic@ihtm.bg.ac.rs; 3 Department of Pharmacology, Clinical Pharmacology and Toxicology, Faculty of Medicine, University of Belgrade, 11000 Belgrade, Serbia; dusan.spasic.dr@med.bg.ac.rs; 4 Institute of Mental Health, Faculty of Medicine, University of Belgrade, 11000 Belgrade, Serbia; cedo.miljevic35@gmail.com

In the original publication [1], there was an error in reference 21. The correct reference 21 appears below.

[21] Minic, S.L.; Milcic, M.; Stanic-Vucinic, D.; Radibratovic, M.; Sotiroudis, T.G.; Nikolic, M.R.; Ćirković Velickovic, T. Phycocyanobilin, a bioactive tetrapyrrolic compound of blue-green alga Spirulina, binds with high affinity and competes with bilirubin for binding on human serum albumin. *RSC Adv*. **2015**, *5*, 61787–61798. https://doi.org/10.1039/c5ra05534b.

This change reflects the author’s (Bobone et al., 2014) remark that the formulated statement does not accurately reflect the conclusions of their study on using synchronous fluorimetry to investigate protein-ligand interactions. In the replacement publication, the use of synchronous fluorescence spectrometry in the mentioned context is explained in detail. The authors state that the scientific conclusions are unaffected. This correction was approved by the Academic Editor. The original publication has also been updated.
